# Off-Season Reproductive Performance of Tsurcana Ewes Under Five Estrous Induction Protocols with Different Hormonal Profiles

**DOI:** 10.3390/biology14091217

**Published:** 2025-09-08

**Authors:** Nicolae Adrian Giurginca, Marioara Nicoleta Caraba, Gabi Dumitrescu, Ioan Pet, Elena Pet, Adrian Sinitean, Delia Hutanu, Ion Valeriu Caraba

**Affiliations:** 1 Doctoral School “Engineering of Vegetable and Animal Resources”, University of Life Sciences “King Mihai I” from Timisoara, Calea Aradului 119, 300645 Timisoara, Romania; nicolae.giurginca@usvt.ro; 2 Faculty of Bioengineering of Animal Resources, University of Life Sciences “King Mihai I” from Timisoara, Calea Aradului, 119, 300645 Timisoara, Romania; gabidumitrescu@usvt.ro (G.D.); ioanpet@usvt.ro (I.P.); valeriucaraba@usvt.ro (I.V.C.); 3 Cellular and Molecular Biology Department, “Victor Babes” University of Medicine and Pharmacy of Timisoara, E. Murgu 2, 300041 Timisoara, Romania; 4 ANAPATMOL Research Center, “Victor Babes” University of Medicine and Pharmacy of Timisoara, E. Murgu 2, 300041 Timisoara, Romania; 5 Faculty of Management and Rural Tourism, University of Life Sciences “King Mihai I” from Timisoara, Calea Aradului, 119, 300645 Timisoara, Romania; elenapet@usvt.ro; 6 Biology Department, Faculty of Chemistry-Biology-Geography, West University of Timisoara, Pestalozzi, 16, 300315 Timisoara, Romania; adrian.sinitean@e-uvt.ro (A.S.); delia.hutanu@e-uvt.ro (D.H.)

**Keywords:** sheep, estrus, fertility, pregnancy, lamb, hormonal treatment

## Abstract

The low profitability of small-scale dual-purpose (milk–meat) farming has caused the decline of Tsurcana sheep, making hormonal reproductive optimization essential to sustain Tsurcana sheep farming. We aimed to identify an effective protocol for out-of-season estrous induction in Tsurcana micro-farms. Five protocols were tested in multiparous ewes under free-range conditions (n = 100; 20/group): T1—prostaglandin F_2_α (PGF_2_α) on day 9/gonadotropin-releasing hormone (GnRH) on day 12; T2—PGF_2_α on day 9/pregnant mare serum gonadotropin (PMSG) on day 11; T3—PGF_2_α + PMSG on day 9/GnRH on day 12; T4—PGF_2_α on day 12/PMSG on day 13; T5—PMSG on day 13. The fluorogestone acetate (FGA) sponges were removed on day 9 (T1–T3), day 12 (T4), or day 13 (T5). The estrous responses (70–100%) mainly occurred 36–48 h post-sponge removal, with no differences between protocols or time points. The sponge losses (≤10%) were similar among groups. The occurrence of vaginitis differed significantly between the groups (*p* = 0.008) and was restricted to the long-duration treatments (T4 and T5; ≤10%). Singleton births predominated, the twinning rate was modest (highest in T1–T3), and no multiple births occurred. The lambing, singleton, twinning, and prolificacy rates, as well as birth weights, showed no significant differences between the groups. The estrous responses correlated strongly with the pregnancy, lambing, and singleton rates (Kendall’s τ = 0.82, *p* < 0.05). Cost analysis identified T1 as the most efficient protocol. Short-duration sponge retention combined with PGF_2_α and GnRH (T1) provides the optimal reproductive results for sustainable Tsurcana micro-farming.

## Simple Summary

Tsurcana sheep are a traditional Romanian breed, but their numbers are declining because small family farms that raise them for both milk and meat often struggle to remain profitable. Supporting the survival of this breed requires improving their reproduction, especially outside the natural breeding season when lambs are needed for income and flock renewal. In this study, five hormone-based methods for inducing estrus, or readiness for mating, were tested in 100 adult ewes kept under free-range conditions. Most ewes showed heat within two days of treatment, and the fertility and lambing results were generally good. No major differences were found between methods, although mild vaginal irritation occurred with the longest treatments. Most births were single lambs, while twins were rare. Shorter treatments offered the best balance of effectiveness, animal health, and cost. These results are valuable for society because they provide small-scale farmers with practical tools to improve flock productivity, preserve a traditional sheep breed, and support the sustainability of rural communities.

## 1. Introduction

The rearing of indigenous sheep breeds has experienced a marked decline during recent decades despite their advantages in terms of resistance to diseases, climatic resilience, and suitability for sustainable farming practices [1]. Examples are the Tsigaie and Tsurcana breeds, which are traditionally raised in Romania for meat (lamb) and milk production. Given the short commercialization window for lamb meat, the modest milk yield during the weaning–estrus interval, and low national subsidies, this mixed meat–milk production system faces major economic challenges, especially small farms. These drawbacks can be counterbalanced by increasing lamb output via assisted reproduction techniques and enhancing milk yield and quality through high-quality feed [2] to optimize reproductive management and farm productivity [3,4,5,6].

The health status of breeding animals is a key determinant of the effectiveness of breeding and assisted reproduction techniques [7,8,9,10]. Different hormonal protocols are used in seasonally anestrous ewes to initiate/synchronize reproductive activity [6,11,12,13,14]. As a general rule, progesterone (and its analogues) and equine chorionic gonadotropin (eCG) (and similar compounds) are used to trigger and induce estrus during the anestrous season: the former extends the luteal phase and ensures the synchronized onset of estrus, while the latter is administered at the end of the treatment to stimulate follicular development and ovulation [8,14,15]. In contrast, prostaglandins are applied during the breeding season when the females still have a functional corpus luteum [5,15,16,17,18,19,20]. However, progesterone remains the key reproductive hormone for controlling estrous cycles in sheep, and is used in both breeding [21,22,23] and non-breeding seasons. Intravaginal sponges are typically preferred in small farms for hormone delivery over controlled internal drug release (CIDR) and injectable preparations due to their low cost, ease of use, effectiveness, and suitability for small flocks [24,25,26]. These sponges are routinely applied in short-term protocols (5–7 days) and long-term protocols (12–14 days), although their prolonged retention increases the risk of vaginitis [14,15,16,27,28].

Gonadotropins, including gonadotropin-releasing hormone (GnRH) and equine chorionic gonadotropin (eCG, also known as PMSG—pregnant mare serum gonadotropin), are widely used for estrous induction and ovulation stimulation. GnRH causes the anterior pituitary to release luteinizing hormone (LH) and reduces the amount of follicle-stimulating hormone (FSH), inducing maturation/ovulation of the dominant follicle. PMSG mimics both LH and FSH, promoting follicle growth and ovulation [14,15,26,29]. The addition of luteolytic agents—mainly prostaglandin F_2_ alpha (PGF_2_α) or its analogues—further improves the efficacy of estrous induction in sheep [15,26]. However, reproductive management in ruminants also relies on controlling ovarian luteolysis via pharmaceutical interventions. Dinoprost tromethamine, a synthetic PGF_2_α analogue, and its analogues are potent luteolytic agents, and when used alone or with progestins, they can improve estrous synchronization [12,14,30,31,32,33]. In the non-breeding season, PGF_2_α ensures luteolysis [7,10] while eCG stimulates follicular growth; when administered together before device withdrawal, they enhance synchronized ovulation and improve fertility.

The optimal estrous induction (and synchronization) protocols differ between breeds. In Ordabasin sheep, intravaginal devices with gestagens were applied in combination with eCG [8]. In Karakul and Romanov sheep, progesterone was co-administered intramuscularly with eCG [6,8]. In Awasi sheep, the use of intravaginal sponges with FGA was coupled with other preparations, such as FGA + PGF_2_α, FGA + PMSG, or FGA + PMSG + PGF_2_α [34]. In Kivircik sheep, intravaginal sponges with FGA were used in conjunction with PMSG [35]. In the new Hu sheep, several treatment protocols were tested, such as FGA + PGF_2_α + PMSG, FGA + PGF_2_α with GnRH, FGA + PGF_2_α + PMSG with GnRH, FGA + PGF_2_α + PMSG, or FGA + PMSG [15]. In contrast, there is limited data on estrous induction in the Tsigaie and Tsurcana breeds. Deac et al. compared the efficiency of intravaginal sponges with FGA in conjunction with PMSG and dinoprost or cloprostenol in Tsigaie sheep [14]. Bogdan et al. induced estrus across breeding and non-breeding periods in the Tsurcana breed using progesterone with PGF_2_α and PMSG. The results were inconclusive, and some treatments were ineffective outside the breeding season [11]. Another study compared two protocols involving progesterone (with PGF_2_α and either PMSG or GnRH) for off-season estrous synchronization in Tsurcana sheep before embryo transfer from Suffolk donors [36]. However, there are no direct comparisons of different hormonal regimens, doses, and durations (under identical conditions). Little data is available on the incidence of sponge loss, vaginitis, or other side effects. Moreover, most studies addressed estrous responses, while data on long-term fertility, lambing rate, and lamb survival are scant.

In this context, this study aimed to identify an effective hormonal regimen for promoting estrus outside the natural breeding season in micro-farms raising Tsurcana sheep. These farms routinely use a free-range production system, with sheep undergoing natural mating. We designed this study to deliver a protocol tailored to small-scale, traditional farming systems and provide a practical approach to optimizing reproduction management in this breed. These data should strengthen both the economic viability and genetic sustainability of Tsurcana sheep. These findings may also contribute to maintaining rural livelihoods and preserving the cultural heritage associated with traditional sheep farming.

## 2. Materials and Methods

### 2.1. Location and Animals

The study was conducted between May 2024 and December 2024 on a private farm (raising Tsurcana sheep) located in western Romania (latitude: 44°57′33.3″ N; longitude: 22°16′28.1″ E) at an altitude of 395 m. It started during the natural anestrous season of this breed, that is, late spring to early summer (May–June). Tsurcana sheep are seasonal breeders, with peak natural mating activity from October to January [14]. To ensure that all the animals included in the study were acyclic, estrus behavior was monitored for two weeks prior to treatment using teaser rams, and no ewes exhibited standing estrus. Farm reproductive records confirmed the absence of recent breeding activity.

The experimental group included 100 sheep, aged 2 to 3 years and with an average weight of 43.90 ± 2.35 kg. The sheep were randomly selected from the entire flock, with all sheep having completed their second/third lambing prior to the study without dystocia or lamb mortality. At the end of May, five rams of the same breed—aged 2–4 years old and each weighing 76.35 ± 3.45 kg—were introduced into the pen with the ewes for four weeks to facilitate natural mating. One ram was assigned to each group, yielding a male-to-female ratio of 1:10, and the mating behaviors were monitored daily. All ewes were naturally mated with fertile rams as per the free-range production system used by the participating micro-farms. All the rams used for natural mating underwent a breeding soundness evaluation prior to the experiment, i.e., measurements of scrotal circumference, assessment of physical soundness, and semen quality analysis (motility, concentration, and morphology). Only rams meeting the established fertility standards were used for breeding. All experimental procedures and animal care conditions complied with the recommendations of European Union Directive 86/609/CEE and were approved by the Institutional Committee for Animal Welfare of the University of Life Sciences “King Mihai I”, Timisoara.

### 2.2. Hormonal Treatments for Estrous Induction

**Figure 1:**
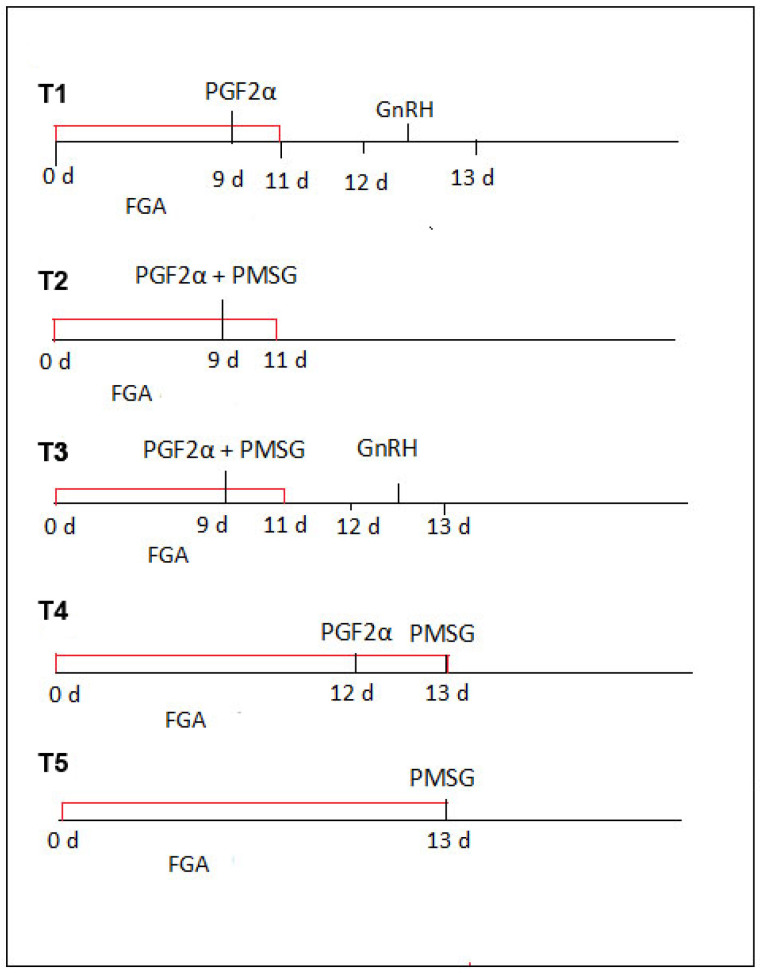
Protocols for administration of hormonal treatments in Tsurcana sheep. PGF_2_α = prostaglandin F_2_α; PMSG = equine serum gonadotropin, GnRH = gonadotropin-releasing hormone; FGA= fluorogestone acetate. T1 = sponge administered for 11 d, PGF_2_α given on d9, GnRH given 36 h post-removal; T2 = sponge administered for 11 d, PGF_2_α + PMSG given on d9, no GnRH; T3 = sponge administered for 11 d, PGF_2_α + PMSG given on d9, GnRH given 36 h post-removal; T4 = sponge administered for 13 d, PGF_2_α given on d12, PMSG given on removal day; T5 = sponge administered for 13 d, PMSG given on removal day, no PGF_2_α or GnRH.

The sheep were divided into five experimental groups and given different hormonal preparations. Details of the protocol used for estrous induction are shown in Figure 1. All the sheep were treated with FGA (45 mg fluorogestone acetate) vaginal sponges (SYNCRITE-45 Vaginal Sponge, Animal Health Supplies, Ascot Vale, VIC, Australia) soaked with a solution of 160 international units (IU) of sodium penicillin to prevent vaginitis. The sponges were inserted into the vagina using the Chronogest CR applicator device (Intervet International, Boxmeer, The Netherlands). The day of sponge insertion was designated as day 0 of estrous induction. After each insertion, the applicator was wiped clean and then placed back in the disinfectant solution (provided with the product pack).

The intravaginal sponges were kept in place for 11 days for the first three experimental groups (T1, T2, and T3) and for 13 days for groups T4–T5, which were withdrawn during the same day. Briefly, the protocols involved the following:

- For T1, 0.1 mg of PGF_2_α (Dinolytic (5 mg dinoprost), Pfizer AG, Zurich, Switzerland) was given intramuscularly (IM) two days before sponge removal; 36 h after removal, 6 µg of gonadotropin-releasing hormone (GnRH; Fertagyl^®^, Merck Animal Health, Rahway, NJ, USA) was administered intramuscularly (IM).
- For T2, on day 9, PGF_2_α (0.1 mg) + PMSG (300 IU) (Sergon, IM; Bioveta, Komenského, Czech Republic), but no GnRH, were given.
- For T3, on day 9, PGF_2_α (0.1 mg) + PMSG (300 IU) were given IM; 36 h after sponge removal, GnRH (6 µg) was given IM.
- For T4, on day 12, PGF_2_α (0.1 mg) was given IM; on the sponge removal day, PMSG (300 IU) was given IM.
- For T5, on the sponge removal day (day 13), only PMSG (300 IU) was given IM.

PGF_2_α and GnRH were applied via IM injection into the rump (gluteal muscles) using a sterile 2 mL syringe and 21 G needle as per the manufacturer’s instructions. The products were administered slowly to minimize tissue irritation. The injection sites were cleaned with 70% ethanol prior to administration, and the hormones were injected slowly to ensure proper intramuscular deposition. All injections were performed by the same experienced operator to ensure consistency, accuracy of dose delivery, and compliance with animal welfare practices.

### 2.3. Reproductive Performance

Following removal of the FGA sponges, the ewes were exposed to fertile rams fitted with marking harnesses (crayon marking system). The ewes showing a fresh crayon mark on the rump were identified as being in estrus. This approach enabled us to visually detect/monitor mating activity within the flock. The presence of a standing reflex in response to teaser (vasectomized) rams was deemed indicative of estrus in hormone-treated ewes. The ratio of teaser rams to test ewes was 1:10. The number of estrous ewes was determined at 24, 36, 48, 60, and 72 h after estrous induction. Assessing the response rate over different time intervals allowed us to establish the timeframe in which the highest proportion of ewes exhibited estrus, which is critical for the efficient timing of natural mating or artificial insemination. This approach ensures that breeding is performed during the peak fertility window. Heat detection commenced immediately after sponge removal for all treatment groups, i.e., starting on day 12 for treatments T1–T3 and day 14 for treatments T4–T5, and continued at regular intervals until the end of the observation period.

During the entire hormonal treatment period, the sheep were monitored to determine the vaginal sponge retention rate and identify the presence of vaginitis. Vaginitis was documented following intravaginal administration of FGA sponges—in line with previous reports indicating that local irritation, mucous discharge, and mild inflammation are common side effects of intravaginal progestagen devices in small ruminants [37]. These reactions are typically attributed to the mechanical friction and alterations in the vaginal microenvironment that occur during sponge retention rather than infectious processes [37]. The onset of vaginitis was detected as changes such as bad odors and/or purulent vaginal discharge. Monitoring for sponge loss and vaginitis onset was conducted at three-day intervals using low-stress techniques (quiet movement, same handlers, and non-slip short race). Routine checks were only performed externally (string presence/length, vulvar discharge score 0–3, and tail soiling); speculum examination was reserved for ewes with abnormal external findings (increased discharge, malodor, fever, or absence of string). All procedures were performed with single-use lubricated gloves and the restraint time was kept under 90 s per ewe. Pregnancy was confirmed via transabdominal ultrasonographic examination with a MiniTube Sonograf (Minitüb GmbH, Bayern, Germany) at about 45 days after estrous induction. The observations were conducted by trained personnel under consistent environmental and nutritional conditions.

These data were used to determine the sponge loss rate (SLR), vaginitis rate (VR), estrous response rate (ERR), and pregnancy rate (PR). The SLR was calculated as the percentage of animals that prematurely lost the intravaginal sponge—usually containing progestogens—before the intended removal date. The vaginitis rate was the percentage of ewes that developed vaginitis. The estrous response rate was calculated as the ratio between the number of ewes exhibiting behavioral estrus and the total number of treated ewes, multiplied by 100. The pregnancy rate was expressed as the percentage of pregnant ewes relative to the total number of synchronized ewes.

As a key reproductive metrics in sheep production, the lambing rate (LR) refers to the percentage of ewes that successfully gave birth post-mating or insemination. We also calculated the singleton lambing rate (SLR), which is the percentage of singleton births among all the ewes that carried to term. In a similar manner, we determined the twinning (lambing) rate (TLR) and multi-lamb rate (MLR) for twin and multi-births, respectively. The prolificacy rate (PPR) refers to the average number of lambs born per ewe and was expressed as the percentage of the total number of lambs born divided by the total number of ewes that gave birth. Finally, we determined the cost of the hormonal drug per ewe treated, and then calculated the cost per lamb born and cost per successful gestation for each experimental group. These values were calculated using the total hormonal drug cost per ewe multiplied by the number of ewes treated in each group, divided by either the total number of lambs born or the total number of pregnancies. All the data were collected by qualified staff under uniform environmental and dietary conditions.

### 2.4. Statistical Analysis

Statistical analysis was performed using Statistica 8 software. The data on estrous induction were analyzed using a Chi-square test of independence to assess the differences in estrous response between groups and time points [37]. Only variables with non-zero values were included in the analysis. Kendal tau correlations were calculated to assess the degree of association between selected reproductive indicators, i.e., estrous response rate, pregnancy rate, lambing rate, singleton lambing rate, and twinning (lambing) rate. This approach allowed us to evaluate whether higher estrous response rates were consistently accompanied by higher pregnancy, lambing, or twinning rates while also accounting for the ordinal nature of the data and the relatively small sample size. The ewes that lost sponges were left with the rams but were excluded from the analysis to avoid bias. Finally, ANOVA was implemented to analyze the datasets related to birth weight after verifying the assumptions of normality and homoscedasticity. In case of significant differences, post hoc comparisons were made using Tukey’s HSD tests. A two-tailed *p* value less than 0.05 was considered significant [38].

## 3. Results

The data on the temporal dynamics of the estrous induction—reflected by the estrous response rate—are shown in Table 1. A robust estrous response was observed between 36 and 48 h post-treatment. Peak estrus expression occurred at 24–36 h post-sponge removal for groups T1 and T3 and at 36–48 h for groups T2, T4, and T5. Interestingly, group T1 exhibited the most uniform distribution of responses across all time intervals. We also note that most estrous responses clustered within the 36–48 h window. Hence, this time frame appears to be the optimal detection period post-treatment. However, the statistical analysis revealed no significant differences in the temporal dynamics of the estrous response between the groups (χ^2^ = 19.14, *p* = 0.261). These findings indicate that the five estrous induction protocols yielded similar results.

Table 2 shows the values of the reproductive management parameters sponge loss rate, vaginitis rate, and estrous response rate. Sponge loss was only observed in the T2–T5 treatment groups. It occurred most frequently in the T5 group. However, the sponge loss rate did not differ significantly between treatment groups (χ^2^ = 6.73, *p* = 0.151). In contrast, the incidence of vaginitis differed significantly between the groups (χ^2^ = 13.61, *p* = 0.008), with cases only occurring in the T4 and T5 groups. These findings indicate that specific procedural or pharmacological aspects of the protocols used in these two groups may predispose ewes to post-treatment infections. With respect to estrous response rate, we identified a complete response for the T1 treatment group, an acceptable response in the T2–T4 groups, and a reduced response for the T5 protocol. However, the estrous response rates were similar across treatments (χ^2^ = 7.74, *p* = 0.102).

Table 3 presents the values of the key reproductive performance metrics: pregnancy rate, lambing rate, singleton lamb rate, twinning rate, multi-lamb rate, and prolificacy rate. The T1 and T3 treatments yielded the greatest pregnancy and lambing rates. The former treatment group also exhibited the highest prolificacy rate, while the T5 protocol yielded the lowest reproductive success rate. The pregnancy rates differed significantly among the five treatment groups (χ^2^ = 13.72, *p* = 0.008). Single births predominated in all groups, particularly in the T1–T2 groups. The twinning rates were modest in all groups, with the highest rate observed with the T3 protocol. In addition, no multiple births were observed during the study. Moreover, there were no significant differences in lambing rate (χ^2^ = 1.52, *p* = 0.822), singleton lambing rate (χ^2^ = 3.34, *p* = 0.502), twinning rate (χ^2^ = 0.66, *p* = 0.955), and prolificacy rate (χ^2^ = 2.40, *p* = 0.661) between the groups.

**Figure 2:**
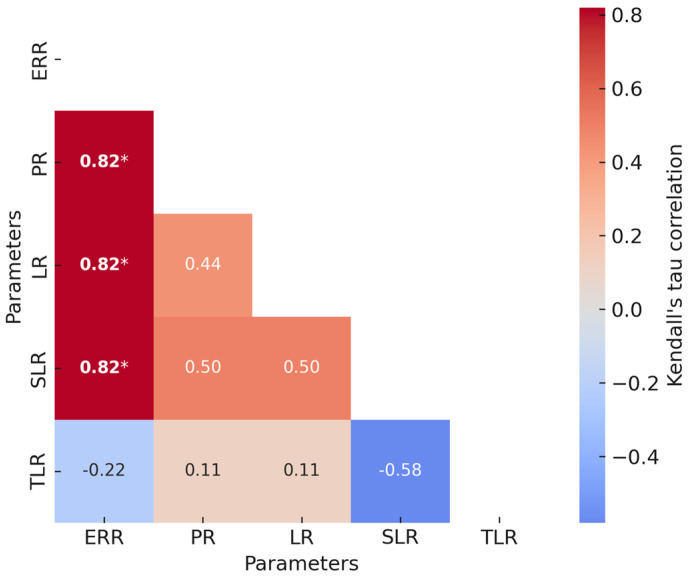
Kendall coefficients for statistical analysis of reproductive parameters after hormonal treatment. ERR = estrous response rate; PR = pregnancy rate; LR = lambing rate; SLR = singleton lambing rate; TLR = twinning (lambing) rate. Bold values annotated with asterisks (*) indicate significant associations (Kendall tau correlations): *—*p* ≤ 0.05.

A heatmap of the Kendall tau correlations is given in Figure 2. The estrous response rate strongly and directly correlated with the pregnancy rate, lambing rate, and singleton lambing rate. However, there were no relationships between the other variables analyzed, including twinning (lambing) rate.

The five experimental protocols yielded 22, 20, 14, 12, and 10 lambs. The birth weights were 3.72 ± 0.61 kg for treatment T1; 3.62 ± 0.52 kg for treatment T2; 3.84 ± 0.53 kg for treatment T3; 4.01 ± 0.29 kg for treatment T4; and 3.74 ± 0.37 kg for treatment T5 (Table S1: Lamb weight at parturition). There were no significant differences in this parameter (ANOVA, F = 0.80, *p* = 0.532) between the groups. As a result, one can conclude that the synchronization protocol did not influence the lamb birth weight. Overall, the T1 and T3 groups showed the best reproductive performance, whereas the T5 group exhibited the weakest results despite full estrus detection. This finding indicates potential post-estrus fertility issues or limitations of this protocol.

The hormonal protocols differed in the cost per treated ewe, per lamb born, and per pregnancy. The protocols incorporating PMSG (T2–T5) and/or GnRH (T1 and T3) displayed higher costs per treated ewe due to the additional hormone expenses. The T1 protocol had the lowest cost per lamb born, followed by T2, whereas T3 and T4 showed higher values due to lower prolificacy and lamb output relative to the hormonal costs. The cost per pregnancy followed a similar pattern, with the T1 treatment again being the most cost-efficient protocol. The high per-pregnancy cost in the T3 treatment group reflects the increased hormonal expenditure from including both PMSG and GnRH without a proportional gain in reproductive success.

## 4. Discussion

This study comes at a moment when small sheep farming with traditional breeds has encountered major problems related to low profitability, rising production costs, animal welfare issues, and climate change [39]. Our work is timely, contributing to our understanding of Tsurcana sheep husbandry. First, we demonstrated the feasibility of applying off-season hormonal treatment schemes to this breed. This expands the reproductive management strategies for locally adapted sheep breeds. Second, our findings support the use of a short sponge retention time to prevent vaginitis and controlled treatment periodicity to protect the female reproductive system. Third, this study aligns with the current trend of promoting sustainable genetic improvement in native sheep breeds via natural mating in free-range flocks and the introduction of valuable rams [38]. These contributions not only address the gaps in the literature on Eastern European sheep breeds, but also have direct applicability to small-scale production systems.

### 4.1. Sponge Loss Rate

The use of progesterone-impregnated sponges in ewes is known to substantially increase reproductive performance [14,40]. Sponge loss may jeopardize this effect by undermining estrous synchronization, reducing pregnancy rates, increasing labor costs, and predisposing ewes to reproductive tract infections. As a result, sheep that lose their intravaginal sponge are typically excluded from experiments. In our study, a low rate of sponge loss was observed—up to 10%—with no statistically significant differences between the groups. Another study investigating estrous induction in Tsurcana sheep found no loss of intravaginal sponges in 60 sheep of similar age undergoing estrous synchronization with dinoprost or cloprostenol [14].

Comparable results were reported for other breeds. For example, Yu and al. found that there were no significant differences in sponge retention rate in 150 healthy multiparous Hu ewes [15]. From both a physiological and mechanical point of view, it is expected that longer intravaginal sponge retention may cause an increase in the rate of sponge loss [41]. In contrast, several studies indicate that intravaginal sponge retention time is not associated with an increased loss rate [15,42]. However, based on the findings of the present study, we believe that extending the duration of the intravaginal sponge-based hormonal treatment beyond 12 days may increase sponge loss.

### 4.2. Vaginitis Rate

The vaginitis rate is routinely monitored during estrous induction and ewes developing this disease are typically removed from breeding programs. There is evidence that sheep subjected to vaginal progestagen therapy tend to develop vaginitis and exhibit changes in their vaginal flora [43,44]. These processes induce histological and cytological changes in the vaginal walls [45]. Since these changes are accompanied by inflammatory processes, treatment with antibiotics is commonly used to prevent vaginitis in sheep, although this approach cannot fully prevent it [46].

In our study, vaginitis was detected exclusively in the T4 and T5 treatment groups, albeit at low rates. These data demonstrate that longer retention of intravaginal FGA-impregnated sponges is associated with the occurrence of vaginitis. Hence, one can expect that the duration of intravaginal sponge retention and the sanitary conditions on the farm are important factors influencing reproductive outcomes in ewes. Indeed, extended retention increases mucosal contact time, potentially increasing bacterial proliferation and the risk of inflammation This association has been previously reported for this sheep breed, with Deac et al. identifying both sponge retention duration and hygiene practices during insertion/removal as contributing factors to vaginitis onset [14].

In fact, short-duration intravaginal sponge protocols—typically 5–7 days or, in some cases, around 9 days—have been specifically developed to reduce the incidence of vaginal pathology while still achieving estrous synchronization in sheep. Shorter insertions result in less vaginitis compared to the conventional 12–14-day sponge programs. The rationale is that a shorter period causes less accumulation of secretions and microbial overgrowth. For example, a 6–9-day sponge regimen is often sufficient to synchronize estrus when combined with appropriate hormonal signals, and it results in only mild vaginal discharge in most ewes. In contrast, extended retention (≥12–14 days) essentially guarantees a higher vaginitis rate [28].

The difference in reproductive outcomes was also striking. Prolonged vaginitis from extended sponge use can negatively impact fertility—likely by creating an inhospitable environment for sperm and embryos. In a comparative trial with 100 Lacaune multiparous sheep (2 to 5 years old), the individuals on a long-term sponge protocol (≈14 days) had a pregnancy rate of only about 45% versus about 70–75% pregnancy rates in ewes on either short-term sponge protocols or using controlled internal drug release (CIDR) devices. The long-term sponge group’s fertility was clearly poorer, correlating with its higher incidence of infection and vaginal lesions. In contrast, short-duration sponge treatments (or those that use CIDR devices) led to pregnancy outcomes comparable to the untreated controls [28]. These findings underscore that extending sponge retention beyond the typical 11–14-day window greatly increases the vaginitis severity and can reduce breeding success. In summary, keeping intravaginal sponges for the minimum effective duration—usually no more than 9–10 days in modern protocols—is a key strategy to prevent severe vaginitis and preserve the reproductive performance of the flock.

### 4.3. Estrous Response Rate

We obtained a satisfactory to excellent estrous response rate, i.e., between 70 and 100%. This reflects an effective synchronization protocol and favorable reproductive performance under practical conditions. Roșca et al. also found estrous response rates of 80–100% in Tsurcana sheep used as recipients for Suffolk embryos [36]. Similar rates were reported for other sheep breeds [47,48,49,50].

The lowest estrous response rate (70%; 14/20) was observed in the T5 group, an obviously lower rate compared to the (near) complete responses (90–100%) observed in the T1–T4 treatment groups. This clinically relevant decrease is in line with farm evidence; that is, prolonged intravaginal sponge retention and the absence of luteolytic/ovulatory support alter estrous synchronization and expression in ewes. Notably, this group likewise exhibited the highest vaginitis rate. It is likely that the 13-day intravaginal sponge retention period increased the incidence of (sub)clinical vaginitis, leading to a lower estrous response. On the other hand, the absence of other hormones, such as PGF_2_α and/or GnRH, could further account for this outcome. For example, the corpus luteum can continue to produce progesterone when PGF_2_α is deficient and/or its activity is suppressed, limiting the capacity of ewes to enter estrus synchronously [51]. Under similar circumstances, the absence of exogenous GnRH may lead to variation in the timing of the preovulatory LH surge, resulting in ovulation asynchrony and lowering the proportion of ewes with detectable estrus behaviors [48].

Finally, we note that the aforementioned protocol resulted in the lowest pregnancy and lambing rates. It is plausible that the reduced estrous response rate was the primary driver of the poor overall reproductive performance of this protocol. Significant intergroup differences in this parameter provide evidence that the protocol—especially the sponge retention duration and hormonal sequence—may have directly affected the reproductive success.

### 4.4. Pregnancy Rate, Lambing Rate (Single, Twin, and Multiple Births), Prolificacy Rate

Among the investigated protocols, the T3 treatment achieved the highest pregnancy rate but showed a lower prolificacy. The combined use of prostaglandin, PMSG, and GnRH appeared to have improved conception by ensuring luteal regression followed by a synchronized ovulation, yet the timing or dose of PMSG may have only stimulated a single dominant follicle rather than multiple follicles. This explains why pregnancy was favored but the twinning rate remained modest. In contrast, the T2 protocol, which relied on PGF_2_α and PMSG without GnRH, yielded a balanced ovulatory response with a moderate twinning rate but slightly lower pregnancy rate, likely due to less tightly synchronized ovulation. The comparison of T1 and T2 further suggests that PMSG supports multiple ovulatory events while GnRH ensures optimal ovulation timing. On the other hand, the T4 and T5 groups—with longer sponge retention times (13 days)—resulted in lower prolificacy and twinning rates despite PMSG administration. The extended progestagen exposure may have suppressed the follicular dynamics more strongly, leaving fewer dominant follicles available at sponge removal. Moreover, the absence of GnRH in T5 likely reduced the synchrony of ovulation, further limiting the reproductive efficiency. Taken together, these findings show that hormonal combinations can optimize estrus induction and conception, but prolificacy is influenced by more complex interactions, including hormone timing, dosage, and sponge duration. Shorter treatments with GnRH support (T1 and T3) appeared to be the most effective for maximizing pregnancy outcomes, while protocols relying solely on PMSG or extended sponge use compromise fertility and prolificacy.

The strong, direct correlations between estrous response rate and pregnancy rate, lambing rate, and singleton lambing rate lend credence to its central role in determining reproductive efficiency. In this context, a higher proportion of ewes exhibiting estrus likely reflects better ovarian activity and ovulation synchronization, which in turn increases the probability of successful fertilization and embryo implantation. This association may also reflect the influence of nutritional and hormonal status, that is, animals with a better metabolic balance tend to display stronger estrus behaviors and sustain embryo development more effectively. Hence, the estrous response rate could serve as a practical early indicator of overall reproductive performance.

The twinning rate is an economically relevant parameter in sheep production, with higher rates of multiple births being generally seen as a desirable outcome. On the other hand, excessive twinning may be associated with greater nutritional requirements, increased management demands, and higher risks of perinatal mortality for ewes. The lack of correlation with other reproductive parameters indicates that the twinning rate may vary independently of estrous response, pregnancy, and lambing outcomes. Variability in ovulation response or protocol sensitivity, among other determinants, may underlie this response. Such variability may stem from genetic differences in follicular dynamics, breed-specific reproductive features, or disparities in the physiological status of the ewes at the time of treatment (e.g., body condition, age, or seasonality of ovarian activity). In addition, subtle variations in the pharmacokinetics or pharmacodynamics of exogenous hormones can alter the degree of luteolysis or follicular recruitment, ultimately influencing the ovulation rate. However, the lack of additional supporting data limits our ability to make firm conclusions.

The observation that GnRH-containing protocols (T1 and T3) yielded higher estrous and reproductive rates differs from the commonly reported effect of GnRH in reducing estrus detectability. More precisely, GnRH induces a pre-ovulatory LH rise that leads to ovulation of the dominant follicle within 12–14 h, thereby shortening the behavioral estrus period and complicating visual detection. This may help explain why GnRH is more often used in fixed-time insemination protocols rather than estrus-detection-based systems. In our study, however, estrus was monitored intensively at short intervals after sponge removal. This approach likely increased the chances of recording estrus before the signs disappeared. Another possibility is that the breed-specific physiology of Tsurcana sheep and the off-season timing of the treatment may have prolonged the expression of estrus.

Although relatively scarce compared to other breeds, the available data on Tsurcana sheep and estrous induction are largely consistent with our findings. Bogdan et al. investigated estrous induction and synchronization in 130 sheep aged 1.3 to 6 years. When administering intravaginal sponges and PMSG, the estrous response, pregnancy, and prolificacy rates were 82.14%, 75%, and 103.57%, respectively. When using vaginal sponges in conjunction with gonadotropins and prostaglandins, the estrous response was 75.56%, the pregnancy rate was 68.89%, and the prolificacy rate was 91.11%. In another study, Ciornei et al. used two protocols for off-season estrous induction and ovulation. The use of FGA-impregnated intravaginal sponges with PGF_2_α and PMSG yielded a 90% estrous response and an 80% ovulation rate. Replacement of PMSG with GnRH resulted in a 60% ovulation rate but a 100% estrous response rate [36], possibly due to the seasonal reproductive capacity of this breed.

### 4.5. Costs Related to the Hormonal Preparations

The T1 treatment showed the lowest costs per sheep, lamb, and pregnancy. It is like that this simplified protocol (using only FGA sponges, a single PGF_2_α injection, and one GnRH dose) contributed to these low costs. In contrast, the T3 treatment generated the highest cost per sheep and pregnancy, showing that additional gonadotropins did not translate into proportional gains in fertility. From a practical perspective, it is important to weigh whether the slightly higher reproductive rates obtained with higher-cost protocols (e.g., T3) justify the additional investment under farm conditions. For small-scale producers, economic efficiency may outweigh marginal gains in pregnancy rate, making the less expensive but effective regimens (such as T1) a more sustainable choice (Table 4).

The other protocols (T2, T4, and T5) exhibited intermediate costs per sheep, while their costs per lamb and per pregnancy were above those seen in T1. This observation reflects a lower efficiency relative to reproductive success. The fact that the T5 treatment (using only PMSG) was economically suboptimal indicates that omitting a luteolysis control (PGF_2_α) or GnRH worsens the cost-to-outcome ratio. Overall, T1 was the best protocol; it merged low drug costs with the best economic efficiency per pregnancy and per lamb. The addition of PMSG and/or GnRH in other protocols increased the cost without proportionally improving the reproductive outcomes, making them less advantageous for small-scale or resource-limited farms.

## 5. Conclusions

This study demonstrated that hormonal protocols for out-of-season estrous induction in Tsurcana sheep differ in their reproductive outcomes, health outcomes, and economic efficiency. Among the five tested regimens, the T1 protocol (FGA sponge administered for 11 days, PGF_2_α given on day 9, and GnRH given 36 h post-removal) achieved the most favorable balance, combining high pregnancy and lambing rates with the fewest complications and the highest economic efficiency. The T3 protocol also achieved high pregnancy rates but with greater economic costs. The T5 protocol (PMSG alone and 13-day sponge retention) showed the weakest reproductive performance and increased the health risks, particularly vaginitis. The estrous response rate was strongly correlated with the pregnancy, lambing, and singleton lambing rates, underscoring its value as a practical indicator of reproductive success in sheep. From an application perspective, the findings confirm that a shorter sponge retention time (11 days) combined with luteolytic and ovulatory hormonal support is optimal for maximizing reproductive efficiency in micro-farms rearing Tsurcana sheep. Adoption of this protocol could enhance both the economic viability and the genetic sustainability of traditional Tsurcana sheep.

## Figures and Tables

**Table 1:** Estrous response rates in different time periods.

Period	T1	T2	T3	T4	T5
	10%	10%	10%	10%	0%
0–24 h	(2/20)	(2/20)	(2/20)	(2/20)	(0/20)
	40%	20%	50%	20%	20%
24–36 h	(8/20)	(4/20)	(10/20)	(4/20)	(4/20)
	20%	40%	20%	40%	40%
36–48 h	(4/20)	(8/20)	(4/20)	(8/20)	(8/20)
	20%	10%	0%	10%	10%
48–60 h	(4/20)	(2/20)	(0/20)	(2/20)	(2/20)
	10%	10%	0%	0%	0%
60–72 h	(2/20)	(2/20)	(0/20)	(0/20)	(0/20)

T1 = sponge administered for 11 d, PGF_2_α given on d9, GnRH given 36 h post-removal; T2 = sponge administered for 11 d, PGF_2_α + PMSG given on d9, no GnRH; T3 = sponge administered for 11 d, PGF_2_α + PMSG given on d9, GnRH given 36 h post-removal; T4 = sponge administered for 13 d, PGF_2_α given on d12, PMSG given on removal day; T5 = sponge administered for 13 d, PMSG given on removal day, no PGF_2_α or GnRH. The measured values are given as percentages with the raw counts in parentheses (number of cases out of the total).

**Table 2:** Reproductive management parameters.

Parameter	T1	T2	T3	T4	T5
SLR	0%	5%	5%	5%	10%
	(0/20)	(1/20)	(1/20)	(1/20)	(2/20)
VR	0%	0%	0%	5%	10%
	(0/20)	(0/20)	(0/20)	(1/20)	(2/20)
ERR	100%	90%	80%	80%	70%
	(20/20)	(18/20)	(16/20)	(16/20)	(14/20)

SLR = sponge loss rate; VR = vaginitis rate; ERR = estrous response rate. T1 = sponge administered for 11 d, PGF_2_α given on d9, GnRH given 36 h post-removal; T2 = sponge administered for 11 d, PGF_2_α + PMSG given on d9, no GnRH; T3 = sponge administered for 11 d, PGF_2_α + PMSG given on d9, GnRH given 36 h post-removal; T4 = sponge administered for 13 d, PGF_2_α given on d12, PMSG given on removal day; T5 = sponge administered for 13 d, PMSG given on removal day, no PGF_2_α or GnRH. The measured values are given as percentages, with the raw counts in parentheses (number of cases out of the total).

**Table 3:** Reproductive performance metrics.

Parameter	T1	T2	T3	T4	T5
PR	90%	88.88%	93.75%	87.50%	85.71%
	(18/20)	(16/18)	(15/16)	(14/16)	(12/14)
LR	100%	88.80%	93.33%	85.71%	83.33%
	(18/18)	(16/18)	(14/15)	(12/14)	(10/12)
SLR	77.77%	75%	57.14%	57.14%	80%
	(14/18)	(12/16)	(8/14)	(8/14)	(8/10)
TLR	22.22%	25%	21.42%	7.14%	10%
	(4/18)	(4/16)	(3/14)	(1/14)	(1/10)
MLR	0%	0%	0%	0%	0%
	(0/18)	(0/16)	(0/14)	(0/14)	(0/10)
PPR	122.22%	125%	100%	85.71%	100%
	(22/18)	(20/16)	(14/14)	(12/14)	(10/10)

PR = pregnancy rate; LR = lambing rate; SLR = singleton lambing rate; TLR = twinning rate; MLR = multi-lamb rate; PPR = prolificacy rate. T1 = sponge administered for 11 d, PGF_2_α given on d9, GnRH given 36 h post-removal; T2 = sponge administered for 11 d, PGF_2_α + PMSG given on d9, no GnRH; T3 = sponge administered for 11 d, PGF_2_α + PMSG given on d9, GnRH given 36 h post-removal; T4 = sponge administered for 13 d, PGF_2_α given on d12, PMSG given on removal day; T5 = sponge administered for 13 d, PMSG given on removal day, no PGF_2_α or GnRH. The measured values are given as percentages with the raw counts in parentheses (number of cases out of the total).

**Table 4:** Cost analysis of estrous induction protocols in sheep (EUR).

Hormonal Drug	T1	T2	T3	T4	T5
FGA	2.75	2.75	2.75	2.75	2.75
PGF_2_α	1.2	1.2	1.2	1.2	1.2
PMSG	0	3.75	3.75	3.75	3.75
GnRH	1.8	0	1.8	0	0
Cost per sheep (EUR)	5.75	7.7	9.5	7.7	7.7
Cost per lamb (EUR)	5.23	6.93	12.67	15.40	11.98
Cost per pregnancy (EUR)	6.39	8.88	10.13	8.80	8.98

FGA = fluorogestone acetate; PGF_2_α = prostaglandin F_2_ alpha; PMSG = pregnant mare serum gonadotropin (equine chorionic gonadotropin, eCG); GnRH = gonadotropin-releasing hormone. T1 = sponge administered for 11 d, PGF_2_α given on d9, GnRH given 36 h post-removal; T2 = sponge administered for 11 d, PGF_2_α + PMSG given on d9, no GnRH; T3 = sponge administered for 11 d, PGF_2_α + PMSG given on d9, GnRH given 36 h post-removal; T4 = sponge administered for 13 d, PGF_2_α given on d12, PMSG given on removal day; T5 = sponge administered for 13 d, PMSG given on removal day, no PGF_2_α or GnRH.

## Data Availability

The data presented in the paper were obtained during research project No. 2542/10.04.2023 and were submitted in the form of a report to the economic agent PFA Giurginca Nicolae Adrian.

## Supplementary Materials

The following supporting information can be downloaded at: https://www.mdpi.com/article/10.3390/biology14091217/s1, Table S1: Lamb weight at parturition.

## Abbreviations

P4	Progesterone
FGA	Fluorogestone acetate
PGF_2_α	Prostaglandin F_2_α
PMSG	Equine serum gonadotropin
MPA	Medroxyprogesterone acetate
GnRH	Gonadotropin-releasing hormone
eCG	Equine chorionic gonadotropin
CIDR	Controlled internal drug release
ERR	Estrous response rate
PR	Pregnancy rate
LR	Lambing rate
SLR	Singleton lambing rate
TLR	Twinning lambing rate
MLR	Multi-lamb rate
PR	Prolificacy
