# Supplementary material for: Off-Season Reproductive Performance of Tsurcana Ewes Under Five Estrous Induction Protocols with Different Hormonal Profiles
Source: Biology (Basel). 2025 Sep 8;14(9):1217. doi: 10.3390/biology14091217 (PMC12467678; doi:10.3390/biology14091217)
Supplement: Supplementary file 1 [file biology-14-01217-s001.zip › biology-3750693-supplementary.pdf]

*Article*

# Evaluation of reproductive performance in Turcana sheep after administration of five estrus synchronization protocols in the non-breeding season

**Nicolae Adrian Giurginca [1,2,†], Marioara Nicoleta Caraba [3,4,*,†], Gabi Dumitrescu [2,4], Ioan Pet [2], Elena Pet [5], Adrian Sinitean [6], Delia Hutanu [6] and Ion Valeriu Caraba [2,4]**

1 Doctoral School "Engineering of Vegetable and Animal Resources", University of Life Sciences "King Mihai I" from Timisoara, Calea Aradului 119, 300645 Timisoara, Romania; nicolae.giurginca@usvt.ro
2 Faculty of Bioengineering of Animal Resources, University of Life Sciences "King Mihai I" from Timisoara, Calea Aradului, 119, 300645 Timisoara, Romania; gabidumitrescu@usvt.ro (G.D.); ioanpet@usvt.ro (I.P.); valeriucaraba@usvt.ro (I.V.C.)
3 Cellular and Molecular Biology Department, "Victor Babes" University of Medicine and Pharmacy of Timisoara, E. Murgu 2, 300041 Timisoara, Romania
4 ANAPATMOL Research Center, "Victor Babes" University of Medicine and Pharmacy of Timisoara, E. Murgu 2, 300041 Timisoara, Romania
5 Faculty of Management and Rural Tourism, University of Life Sciences "King Mihai I" from Timisoara, Calea Aradului, 119, 300645 Timisoara, Romania; elenapet@usvt.ro
6 Biology Department, Faculty of Chemistry-Biology-Geography, West University of Timisoara, Pestalozzi, 16, 300315 Timisoara, Romania; adrian.sinitean@e-uvt.ro (A.S.); delia.hutanu@e-uvt.ro (D.H.)
* Correspondence: nicoleta.caraba@umft.ro
† These authors contributed equally to this work.

**Table S1.** Lambs weight at parturition.

| Parameter | Group I | Group II | Group III | Group IV | Group V |
|---|---|---|---|---|---|
| L 1 | 4219 | 4197 | 4408 | 4360 | 4297 |
| L 2 | 4343 | 3504 | 4525 | 3548 | 4132 |
| L 3 | 4507 | 3425 | 3541 | 3890 | 3863 |
| L 4 | 3658 | 4109 | 4264 | 4136 | 4590 |
| L 5 | 3290 | 3913 | 3982 | 3667 | 4194 |
| L 6 | 3159 | 4190 | 4184 | 3949 | 2991 |
| L 7 | 3254 | 2803 | 4532 | 4128 | 3360 |
| L 8 | 4565 | 3871 | 3606 | 4423 | 3213 |
| L 9 | 3578 | 3108 | 2973 | | 2986 |
| L 10 | 4030 | 3522 | 3321 | | |
| L 11 | 3484 | 2855 | 3165 | | |
| L 12 | 4021 | 3444 | 3681 | | |

| | | | | | |
|---|---|---|---|---|---|
| **L 13** | 4520 | 4264 | | | |
| **L 14** | 3565 | 4320 | | | |
| **L 15** | 3786 | 2984 | | | |
| **L 16** | 4518 | 3218 | | | |
| **L 17** | 3154 | 2965 | | | |
| **L 18** | 2892 | 3153 | | | |
| **L 19** | 2282 | 4236 | | | |
| **L 20** | 3238 | 4470 | | | |
| **L 21** | 3505 | | | | |
| **L 22** | 4215 | | | | |
| **TOTAL LAMBS** | **22** | **20** | **12** | **8** | **9** |
